# Dsg2 arrhythmogenic cardiomyopathy is accompanied by neocortical synaptic changes in adult mice

**DOI:** 10.1186/s13041-026-01355-6

**Published:** 2026-11-11

**Authors:** Amelie Eichler, Josefine Biermann, Sandra A. Hemkemeyer, Jan Hegermann, Camilla Schinner, Maximilian Lenz

**Affiliations:** 1 Hannover Medical School, Institute of Neuroanatomy and Cell Biology, Carl-Neuberg-Straße 1, 30625 Hannover, Germany; 2 Hannover Medical School, Institute of Functional and Applied Anatomy, Carl-Neuberg-Straße 1, 30625 Hannover, Germany; 3 Hannover Medical School, Research Core Unit Electron Microscopy, Carl-Neuberg-Straße 1, 30625 Hannover, Germany; 4 Department of Biomedicine, Section Anatomy, University of Basel, Pestalozzistrasse 20, Basel, 4056 Switzerland; 5 Institute of Anatomy and Experimental Morphology, University Medical Center Hamburg-Eppendorf, Martinistraße 52, 20246 Hamburg, Germany; 6 Hamburg Center for Cardiovascular Research (DZHK) and Hamburg Center for Cardiovascular Research (HCCR), University Medical Center Hamburg-Eppendorf, Hamburg, Germany; 7 Department of Cardiology and Department for BioMedical Research, Inselspital University Hospital Bern, University of Bern, Murtenstrasse 24, 3008 Bern, Switzerland

**Keywords:** Heart failure, Arrhythmogenic cardiomyopathy, Synapse, Synaptic vesicles, Postsynaptic density, Spine apparatus organelle

## Abstract

Heart failure is frequently accompanied by cognitive impairment, yet structural correlates in the brain remain incompletely defined. Here, we assessed whether chronic heart failure is associated with synaptic ultrastructural remodeling in the murine neocortex using a mouse model of arrhythmogenic cardiomyopathy caused by a point mutation in desmoglein-2 (Dsg2-W2A). Brains from aged adult homozygous Dsg2-W2A mice and age-matched wildtype controls were processed for transmission electron microscopy, and excitatory synapses in layer I of the prefrontal cortex were quantitatively assessed, with comparative analyses in the somatosensory cortex. In the prefrontal cortex, presynaptic terminal area and synaptic vesicle number were reduced in mutant mice, whereas postsynaptic measures were not altered. The prevalence of mitochondria and the spine apparatus within synapses was comparable between genotypes, suggesting no major loss of synapse-associated organelles. Finally, synaptic parameters in the prefrontal cortex did not show a systematic dependence on distance to local blood vessels. Together, these findings indicate moderate region- and compartment-specific synaptic ultrastructural alterations associated with Dsg2 cardiomyopathy and chronic heart failure, with predominant effects on presynaptic terminals.

**Supplementary information:**

The online version contains supplementary material available at https://doi.org/10.1186/s13041-026-01355-6.

## Introduction

Heart failure (HF) is a major health burden in the aging population, affecting approximately 1–2% of adults and increasing in prevalence with advancing age [1]. HF is frequently accompanied by cognitive impairment, including deficits in memory and attention [2], correlating with higher risks of hospitalization and mortality. However, the underlying structural correlates of cognitive decline in the context of HF remain poorly understood.

The pathophysiology of HF can be studied using various mouse models based on myocardial ischemia or genetic modifications, resulting in right, left, or global heart dysfunction. A recent study has established a cardiomyopathy model bearing a mutation in the desmosomal cadherin gene desmoglein-2 (Dsg2) [3]. Here, disruption of cardiomyocyte cell-cell adhesion via a Dsg2 point mutation – W2A, abolishing the tryptophan swap *trans*-binding mechanism – causes arrhythmia, biventricular fibrosis, and dilatation. This leads to HF with reduced left ventricular ejection fraction starting from around 6 months of age, mirroring human arrhythmogenic cardiomyopathy [3, 4]. While the mechanisms of ventricular dysfunction and remodeling are under investigation, little is known about concomitant changes in the central nervous system that might contribute to HF-associated cognitive decline [5].

Synapses form highly specialized connection sites between neurons and are crucial for information encoding and cognitive function. Synaptic plasticity – the activity-dependent modification of synaptic strength – is the basis for learning and memory. This adaptive process requires synaptic integrity and the availability of local cellular resources. Here, mitochondria within presynaptic terminals and dendritic spines provide ATP and buffer calcium to meet high energy demand and maintain calcium homeostasis during synaptic transmission [6, 7]. Another critical organelle for synaptic plasticity is the spine apparatus, a specialized stack of smooth endoplasmic reticulum present in a subset of dendritic spines [8]. The spine apparatus is thought to regulate calcium dynamics and protein synthesis in spines [9]. The absence or dysfunction of synaptic resources is associated with alterations in cognitive performance [10, 11].

Given the link between HF and cognitive dysfunction and the importance of synaptic integrity for cognition, we hypothesized that chronic HF might induce synaptic alterations in the brain. In this study, we therefore used transmission electron microscopy (TEM) to examine the neocortical synapses of aged adult Dsg2 mutant mice with advanced HF. We quantitatively assessed ultrastructural features of both pre- and postsynaptic compartments in layer I of the prefrontal and the somatosensory cortex, where the apical dendritic trees of layer II/III principal neurons extend. Moreover, we evaluated the prevalence of mitochondria and the spine apparatus organelles within dendritic spine synapses.

## Materials and methods

### Ethics statement

All mouse experiments were carried out according to the protocol approved by the Cantonal Veterinary Office of Basel-Stadt (License number 2973_32878) and complied with the ARRIVE guidelines. Every effort was made to minimize pain or distress of animals.

### Mouse model

The B6.*Dsg2*^*tm1(W2A)Spin*^ mouse line (Dsg2-W2A) was provided by Volker Spindler, University of Basel, Switzerland. The mouse line was generated by Cas9/CRISPR base editing and the characterization of the heart phenotype was described previously [3]. All mice were housed under specific pathogen-free conditions with standard bedding, chow and drinking water *ad libitum* with 12 h light/12 h dark cycle according to institutional guidelines. For genotyping, DNA was extracted from biopsies in 25 mmol/l NaOH and 0.2 mmol/l EDTA at 98 °C for 1 hour and neutralized with 40 mmol/l Tris pH 5.5. PCR was performed using GoTaq G2 (M7845, Promega, Madison, WI, USA) according to the manufacturer’s instructions with the primers Dsg2-W2A for: GAATGTCTCCCCAAAGCTTTGGGTATG and Dsg2-W2A rev: CTGCTACCTTGGCAATCGGGTTC, which span the mutated region. The PCR product was digested with 66.7 U/ml AluI (R0137, New England Biolabs, Ipswich, MA, USA) in CutSmart buffer (New England Biolabs) overnight at 37 °C. Subsequent electrophoresis detected the Dsg2-WT 197 bp fragment, while the Dsg2-W2A mutant allele was cut into a 109 bp and a 72 bp fragment. For each genotype, 3 animals were included (2 females and 1 male per group).

### Perfusion and organ collection

Mice in the age range of 76–87 weeks were euthanized via intraperitoneal pentobarbital overdose according to guidelines of the Cantonal Veterinary Office of Basel-Stadt and the University of Basel. After confirmation of loss of reflexes, a lateral thoracotomy was performed followed by vascular perfusion via cannulation of the apical left ventricle with opening of the superior vena cava. Subsequent to perfusion of 30 ml phosphate-buffered saline, 20 ml of fixative (1.5% glutaraldehyde and 1.5% paraformaldehyde in 0.15 M HEPES buffer, pH 7.35) was perfused at a hydrostatic pressure of 60 mmHg. Organs including the brain were collected, immersed in fixative and stored at 4 °C until further processing (maximum storage time: 3 weeks). For Sirius Red staining, hearts of mice were extracted, washed in HBSS and fixed in 4% paraformaldehyde in PBS overnight. Hearts were embedded in paraffin with subsequent sectioning at 4 µm thickness following standard procedure. Sirius Red staining was performed to label collagen fibers in red, cytoplasm of cells in yellow and nuclei in brown as described before [3, 12]. Images were acquired with a 20x objective on an AxioScan Z1 slide scanner (Zeiss, Jena, Germany).

### Sample preparation for electron microscopy

After fixation, samples were washed 5 times in 0.15 M HEPES buffer (pH 7.35) for 8 min followed by incubation for 6 times in 0.1 M cacodylate buffer (CB, pH 7.35) for 8 min. Osmification was performed in CB containing 1% osmiumtetroxide for 3 h, followed by 5 washing steps in CB for 8 min and 6 washing steps for 8 min in water. Subsequently, samples were incubated in aqueous 1% uranyl acetate overnight and then washed 5 times for 10 min in water. Dehydration was performed in acetone with the following steps: 70% 4 × 15 min; 90% 4 × 15 min; 100% dry acetone 3 × 15 min and once 2.5 h. Infiltration in epoxy resin (Agar100 kit, Agar Scientific, Wetzlar, Germany, mixture “hard”) was conducted over night in 50% resin in acetone and 24 h in 100% resin, with two exchanges in between. Samples were polymerized at 40 °C overnight followed by 80 °C for 2 days. For defining neocortical regions of interest for TEM, semithin sections were prepared (1 µm). Representative images of the location of the respective cortical areas are shown in Supplementary Figure S2. Ultrathin sections (60 nm) of the selected areas were poststained with uranyl acetate and lead citrate [13].

### Transmission electron microscopy (TEM)

Ultrathin sections were observed with a Morgagni 268 Transmission Electron Microscope (FEI, Eindhoven, Netherlands) and images were acquired with a Veleta CCD camera (Olympus SIS, Muenster, Germany) at a resolution of 2048 × 2048 or 1024 × 1024 pixels. Superficial cortical layers were visually identified at low magnification, and the region of interest was defined as cortical tissue surrounding vessels. For analysis of the prefrontal cortex at least 3 regions of interest in layer I for each mouse were defined and analyzed. For analysis of the somatosensory cortex, 5 regions of interest were defined and analyzed in layer I for each mouse. Several overlapping images were acquired at 22,000 × magnification. Images were saved as .tif files.

### Quantification and statistics

**Fig. 1:**
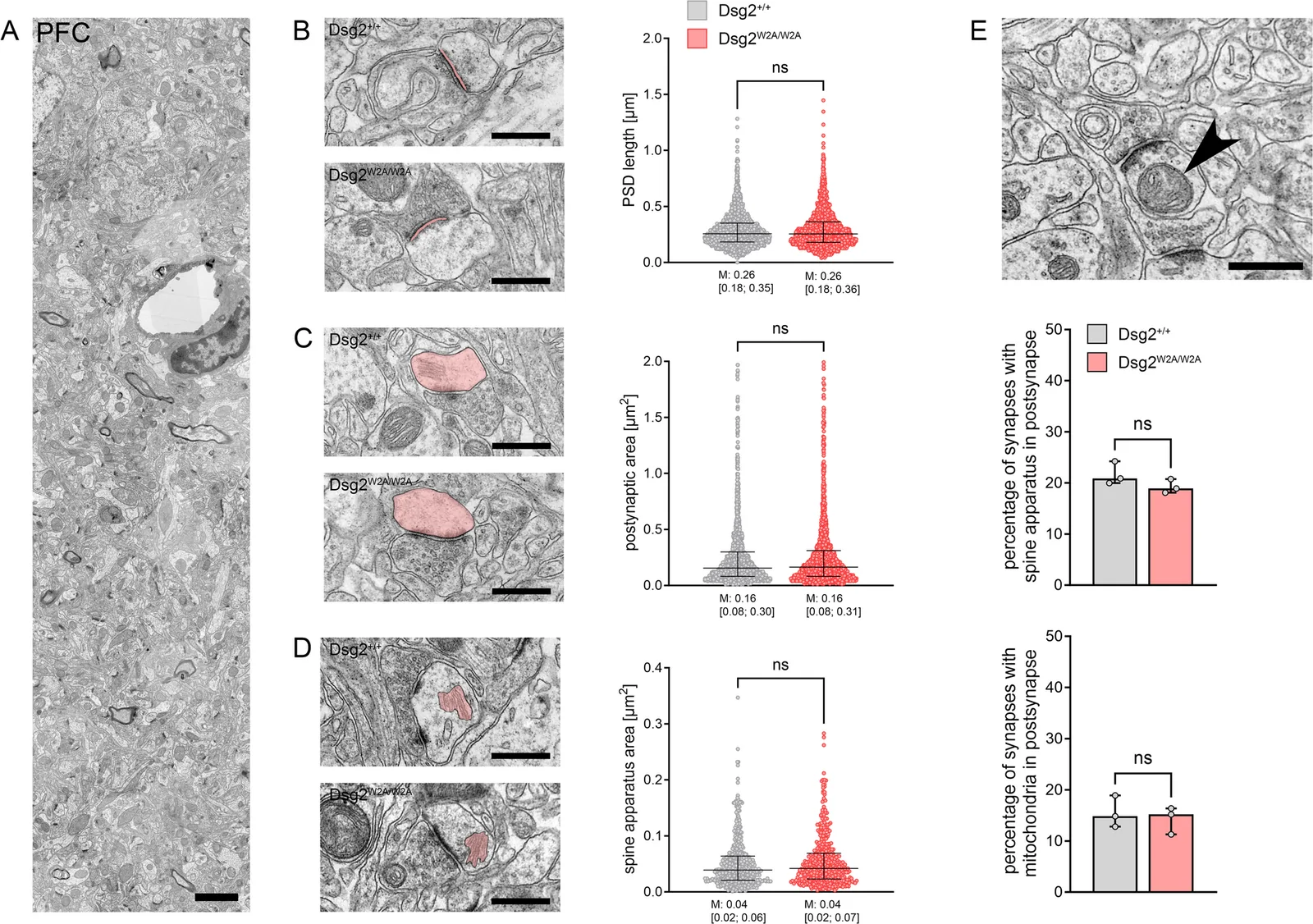
Dsg2-arrhythmogenic cardiomyopathy with HF does not change postsynaptic properties in the prefrontal cortex. (**A**) TEM sample image of brain tissue around a vessel in the superficial layers of the prefrontal cortex of a WT (Dsg2^+/+^) animal. PFC, prefrontal cortex. Scale bar, 2 µm. (**B**)-(**D**) Sample images and group data of PSD length (**B**), postsynaptic area (**C**) and spine apparatus area (**D**) in the superficial layers of the prefrontal cortex in WT (Dsg2^+/+^) and Dsg2 mutant mice (Dsg2^W2A/W2A^). Analyzed parameters are indicated in the representative images with a red overlay. For the group data, median (M) together with the first and third quartiles (in brackets) are provided for each group (PSD length (**B**): n_WT_ = 2352 synapses in three animals; n_W2A_ = 2369 synapses in three animals; postsynaptic area (**C**): n_WT_ = 2352 synapses in three animals, n _W2A_ = 2369 synapses in three animals, 74 data points are outside the axis limits; spine apparatus area (**D**): n_WT_ = 504 synapses in three animals, n_W2A_ = 457 synapses in three animals). Scale bars, 500 nm. (**E**) Upper panel: Representative image of a synapse with a mitochondrion in the postsynaptic compartment (indicated by the black arrowhead). Quantification of synapses with the presence of a spine apparatus organelle (middle panel) or mitochondria (lower panel) in the postsynaptic compartment (n_WT_ = 3 animals, n_W2A_ = 3 animals). Scale bar, 500 nm. Scatter plots: Mann-Whitney test. Bar graphs: Unpaired t-test. Colored dots represent individual data points. Error bars represent median ± interquartile range. ns, no statistically significant difference

Overlapping images were stitched using the semiautomatic function Photomerge and Autostitch of Adobe Photoshop (Version CS2). Morphological assessment was conducted by a researcher blinded to the genotype of the sample. Excitatory synapses that were completely displayed on one stitched image (intact presynaptic and postsynaptic compartment) were morphologically assessed. Morphological assessment was manually performed using the Fiji software [14]. For each synapse, compartments were defined as regions of interest (ROIs). For the analysis of areas, the respective compartment was defined using the Freehand selection tool. The length of postsynaptic densities (PSD length) was analyzed with the Freehand line tool. Vesicles were counted manually using the Multi-point tool. For each analyzed synapse, the presence of mitochondria or the spine apparatus organelle in the synaptic compartment was documented (for representative images see Fig. 1). Spine apparatus organelles were identified by their characteristic structure defined by stacked endoplasmic reticulum with dense protein material between the cisternae (dense plates). The distance to the closest vessel was defined by the minimal distance between the middle of the respective PSD and the lumen of the vessel.

In the sections of the prefrontal cortex of wildtype animals (2 female animals, 1 male animal), from each animal 3 regions of interest in layer I were defined, resulting in a total number of 2352 analyzed synapses. In the sections of the prefrontal cortex of homozygous Dsg2-W2A mutant animals (2 female animals, 1 male animal), from each animal 3 regions of interest in layer I were defined, resulting in a total number of 2369 analyzed synapses.

For the preliminary analysis of the somatosensory cortex, 5 regions of interest of each animal (wildtype: 1 female; Dsg2-W2A mutant: 1 female) were defined, resulting in a total number of analyzed synapses of 1426 in the wildtype animal and 1408 in the Dsg2-W2A mutant animal.

**Fig. 3:**
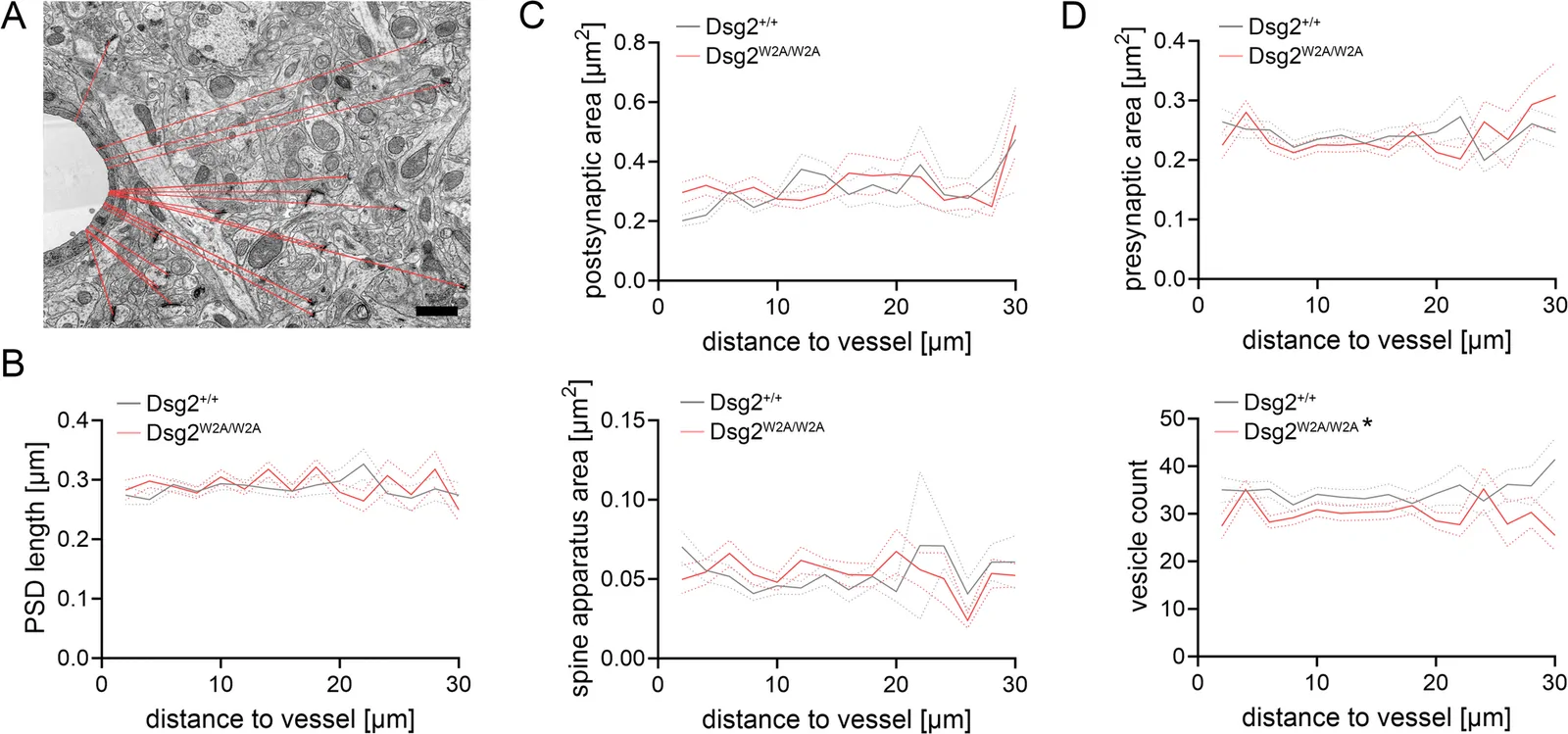
Synaptic morphologies in the prefrontal cortex in relation to their distance to blood vessels. (​**A**) Representative image of brain tissue around a vessel in the superficial layers of the prefrontal cortex. Red lines indicate the distance of the respective synapse to the vessel. Scale bar, 1 µm. (**B**)-(**D**) X-Y graphs show mean values of morphological parameters of synapses in the superficial layers of the prefrontal cortex of wildtype (Dsg2^+/+^) and Dsg2 mutant mice (Dsg2^W2A/W2A^) sorted by their distance to the closest vessel (PSD length, postsynaptic area, presynaptic area, vesicle count: n_WT_ = 2352 synapses in three animals, n_W2A_ = 2369 synapses in three animals; spine apparatus area: n_WT_ = 504 synapses in three animals, n_W2A_ = 457 synapses in three animals). Values were binned in steps of 2 µm (e.g. 0–2, 2–4 µm etc.). For none of the analyzed parameters, the distance to the vessel significantly affects the result of the curve. However, the genotype has a significant effect on the curve progression of vesicle count. Two-way ANOVA. Colored lines represent mean values, error bars show ± SEM. The genotype (column factor) significantly affected the curve characteristics of the vesicle count (*p < 0.0001)

**Fig. 2:**
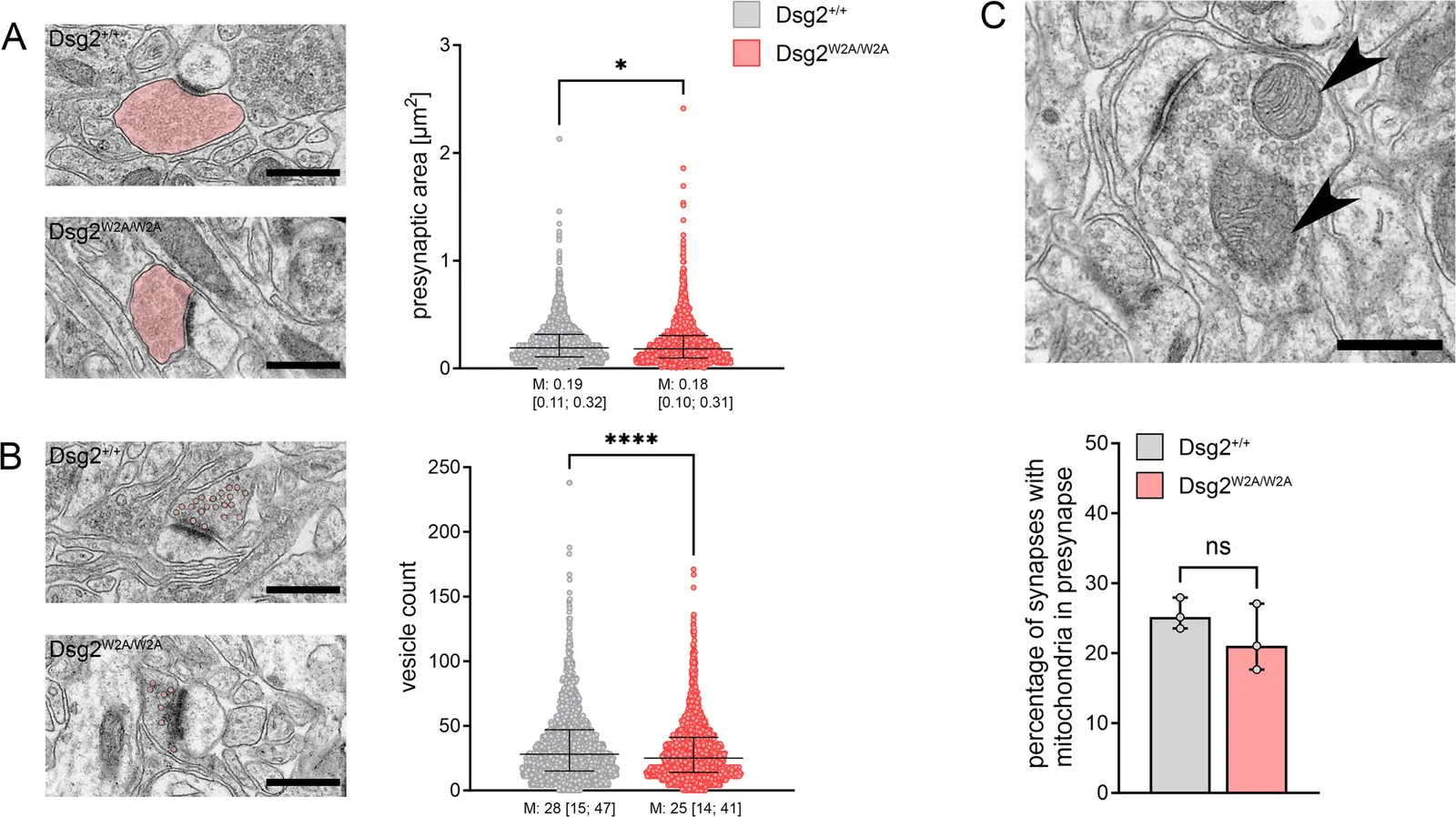
Dsg2 cardiomyopathy alters presynaptic properties in the prefrontal cortex.(**A**)-(**B**) TEM sample images and group data of presynaptic area (**A**) and synaptic vesicle count (**B**) In the superficial layers of the prefrontal cortex in WT (Dsg2^+/+^) and Dsg2 mutant mice (Dsg2^W2A/W2A^). Analyzed parameters are indicated in the representative images with a red overlay. for the group data, median (M) together with the first and third quartiles (in brackets) are provided for each group (n_WT_ = 2352 synapses in three animals, n_W2A_ = 2369 synapses in three animals). Scale bars, 500 nm.(**C**) Upper panel: representative image of a synapse with mitochondria in the presynaptic compartment (indicated by black arrowheads). Quantification of synapses with mitochondria present in the presynaptic compartment (n_WT_ = 3 animals, n_W2A_ = 3 animals). Scale bar, 500 nm. Scatter plots: mann-whitney test. Bar graphs: unpaired t-test. Colored dots represent individual data points. Error bars represent median ± interquartile range. ns, no statistically significant difference; *p < 0.05; ****p < 0.0001

Statistical analysis and visualization were performed using GraphPad Prism 7 and 9 (GraphPad Software). For statistical comparisons of synapses nonparametric tests were used, since normal distribution of data could not be assured. For column statistics, the Mann-Whitney U test (comparison of two groups) was used. For the analysis of X-Y plots (Fig. 3), a Two-Way ANOVA was performed. For comparisons across animals (Figs. 1E, 2C), an unpaired t-test was used. *p* values smaller than 0.05 were considered a significant difference. For column statistics: * *p* < 0.05, ** *p* < 0.01, **** *p* < 0.0001 and not significant differences are indicated by “ns”. For X-Y plots: *p < 0.0001.

### Digital illustrations

Figures were prepared using the Photoshop graphics software (Adobe, San Jose, CA, USA). Image brightness and contrast were adjusted and applied to the entire image.

## Results

### Dsg2-associated cardiomyopathy with chronic HF does not affect postsynaptic ultrastructural properties in the prefrontal cortex of adult mice

To evaluate the influence of HF on the synaptic structure of the central nervous system, we collected brains of homozygous Dsg2-W2A mutant mice (Dsg2^W2A/W2A^), which develop a progressive arrhythmogenic cardiomyopathy phenotype with severe reduction of right and left ventricular output function in echocardiography measurements in mice older than 6 months [3, 15]. Representative images of a Sirius Red stain to visualize biventricular fibrosis in Dsg2-W2A mutant mice (Dsg2^W2A/W2A^) are shown in Supplementary Figure S1. To analyze the influence of chronic HF, brains from mutant and sex- and age-matched wildtype animals were collected and processed for ultrastructural analysis. At first, we analyzed ultrastructural features of excitatory synapses in layer I of the prefrontal cortex (Fig. 1A, for neocortical location see Fig. S1, [30, 23]), focusing on the postsynaptic aspect including the length of postsynaptic densities (PSD length), postsynaptic area, and the area of the spine apparatus organelle (Fig. 1B–D). None of these parameters - postsynaptic area, PSD length, or spine apparatus area - differed between Dsg2 mutant mice and wildtype controls (Fig. 1B–D). We then examined the abundance of synaptic resources, specifically the spine apparatus organelle and mitochondria, in neocortical tissue from mutant and control animals (Fig. 1E). The fraction of excitatory synapses containing a spine apparatus organelle or mitochondria was comparable between Dsg2 mutant and wildtype mice (Fig. 1E). Thus, we conclude that Dsg2-associated cardiomyopathy with chronic HF is not accompanied by ultrastructural postsynaptic alterations in the prefrontal cortex of adult mice, that can be detected via TEM.

### Dsg2-associated cardiomyopathy with chronic HF is accompanied by presynaptic ultrastructural changes in the prefrontal cortex of adult mice

We next assessed ultrastructural features of presynaptic compartments in layer I of the prefrontal cortex, focusing on presynaptic area and vesicle counts (Fig. 2A,B). Both presynaptic area and vesicle number were significantly reduced in Dsg2-W2A mutant mice compared to wildtype controls (Fig. 2A,B). We additionally quantified mitochondrial abundance within presynaptic compartments (Fig. 2C), which did not differ between genotypes. We conclude that Dsg2-associated cardiomyopathy is associated with discrete presynaptic alterations in the prefrontal cortex, indicating a compartment-specific manifestation of chronic heart-failure-related synaptic defects.

### Dsg2-associated cardiomyopathy is accompanied by both pre- and postsynaptic ultrastructural changes in the somatosensory cortex of adult mice

We extended our analysis to another neocortical region, the somatosensory cortex, in adult mice (Fig. S3). As above, we examined ultrastructural features of excitatory synapses in layer I to identify cardiomyopathy-related changes at synaptic sites (Fig. S3A, for neocortical location see Fig. S2). In the somatosensory cortex, the analyzed Dsg2-W2A mutant mouse exhibited a significant reduction in the number of presynaptic vesicles compared to the wildtype control, while presynaptic area remained unchanged (Fig. S3B,C). In addition, PSD length was increased in the Dsg2-W2A mutant mouse (Fig. S3D,E). By contrast, postsynaptic area and the area of the postsynaptic spine apparatus organelle did not differ between genotypes (Fig. S3E). We therefore conclude that, in the somatosensory cortex, Dsg2 cardiomyopathy with HF is associated with presynaptic changes similar to those observed in the prefrontal cortex and accompanied by only minor postsynaptic alterations.

### Synaptic changes in the prefrontal cortex do not depend on the distance to blood vessels

Cardiomyopathy is associated with systemic hypoperfusion and elevated circulating pro-inflammatory cytokines [16, 17]. We therefore hypothesized that cardiomyopathy might differentially affect synapses depending on their distance to local blood vessels. To test this, we quantified pre- and postsynaptic features in the prefrontal cortex and correlated them with the distance to the nearest visible blood vessel in electron micrographs (Fig. 3A). We detected no distance-dependent differences in PSD length (Fig. 3B), postsynaptic area, or spine apparatus area (Fig. 3C) in mutant mice relative to wildtype controls. Likewise, no distance-dependent changes in presynaptic area were observed (Fig. 3D). Notably, the reduction in presynaptic vesicle number in Dsg2 mutant mice did not correlate with distance to blood vessels (Fig. 2D). This indicates that cardiomyopathy-induced synaptic alterations in the prefrontal cortex are independent of local vascular proximity.

## Discussion

In this study, we investigated synaptic ultrastructure in the neocortex of a mouse model of chronic heart failure caused by a Dsg2-W2A mutation. Our findings indicate that Dsg2 cardiomyopathy is accompanied by discrete synaptic alterations in layer I of the neocortex. In the prefrontal cortex, aged mutant mice with chronic HF showed a reduction in presynaptic terminal size and a lower number of synaptic vesicles compared to wildtype controls. Postsynaptic morphology, specifically postsynaptic area and postsynaptic density length, remained unchanged in the prefrontal cortex of mutants. In contrast, the somatosensory cortex exhibited both pre- and postsynaptic changes: The Dsg2-W2A mutant mouse had fewer presynaptic vesicles, despite no change in presynaptic terminal size, and displayed significantly longer PSDs, relative to the wildtype mouse. Given a substantial interdependency of pre- and postsynaptic features, changes in PSDs might partially compensate for alterations in presynaptic terminals and vice versa. Notably, these changes did not depend on the distance to blood vessels. We also assessed key organelles that support synaptic function. The prevalence of the spine apparatus organelle and mitochondria in excitatory synapses was similar between Dsg2-W2A mutants and controls. Taken together, these results suggest that chronic heart failure due to the Dsg2-W2A mutation leads to synaptic remodeling in the neocortex with presynaptic compartments preferentially affected in the prefrontal cortex and combined pre- and postsynaptic ultrastructural changes in the somatosensory cortex.

Synaptic integrity is crucial for normal brain function and cognition [18]. Synapses are highly specialized structures whose size, shape, and molecular composition are dynamically regulated by neural activity, which is tightly linked to learning and memory [19]. In various neurodegenerative and neuropsychiatric conditions, synaptic structural changes are strongly linked to cognitive impairment. In Alzheimer’s disease, synapse loss is one of the main pathological correlates of cognitive decline [20].

While our study reveals significant ultrastructural synaptic differences in Dsg2-W2A mutant mice, there are some limitations to consider. Although a considerable number of synapses and ultrastructural features were analyzed to capture synapse heterogeneity, the number of animals analyzed was relatively small, which may limit the generalizability of our conclusions. Subtle differences might not have reached statistical significance due to limited power. Additionally, some of the observed effect sizes for synaptic changes were modest. Such small effect sizes could correspond to the known heterogeneity in cognitive outcomes among heart failure patients, who show a spectrum from mild cognitive impairment to severe dementia. It is possible that only a subset of synapses or circuits are vulnerable, leading to variable degrees of impairment. Furthermore, key aspects of synaptic ultrastructure were analyzed via TEM in 2D. With 3D electron microscopy, complementary aspects of synaptic composition could be further elucidated (e.g. PSD area, spatial variability in PSD thickness, and spine morphology). Our study focuses on the ultrastructure of excitatory synapses. Although a strong relationship between synaptic structure and function has been established [21], structural alterations might be functionally compensated. Therefore, our conclusions concerning the function of these synapses remain limited. Furthermore, other synaptic entities, such as inhibitory (symmetric) synapses, might represent another target of the disease, with considerable vulnerability in early disease stages and hypoxic states [22, 23]. Importantly, Dsg2 mutation itself is unlikely to directly affect neurons, as Dsg2 expression is not abundant in neocortical neurons of the murine brain [24]. In the brain, Dsg2 is expressed in the choroid plexus which consists mainly of a specialized epithelium and immune cells. Dsg2 mutation could lead to functional changes in choroid plexus function and subsequent secondary effects in neurons. Across at all tissues, Dsg2 is predominantly found in cardiomyocytes and epithelia (Human Protein Atlas [25, 26]. Pathological assessment of different organs of the Dsg2-W2A mouse model revealed a dominant cardiac phenotype resembling the features of arrhythmogenic cardiomyopathy. Consistent with the predominantly cardiac manifestation of arrhythmogenic cardiomyopathy in patients, Dsg2-W2A mice showed no overt pathological changes in the skin, intestine, liver, or lungs at baseline [3]. Therefore, we hypothesize that synaptic alterations in mutant mice are most likely secondary to chronic heart failure.

Future research will have to address the pathophysiology of heart failure-induced synaptic dysfunction. One key area to explore is the role of the blood-brain barrier and brain perfusion in mediating heart-brain interactions [27]. Heart failure is known to reduce cerebral blood flow and can lead to chronic cerebral hypoperfusion and periods of lower oxygen availability in brain tissue. Although synaptic alterations were not dependent on blood-vessel distance, hypoxic stress could impair the metabolism of neurons and synapses, potentially leading to the loss of synaptic resources or structural reorganization. In addition, heart failure is accompanied by a systemic pro-inflammatory state and elevated circulating stress hormones. It should be addressed in further studies how these factors contribute to synaptic changes and cognitive dysfunction. Another important direction will be to map the brain regions and cell types affected by heart failure in more detail. Our study focused on the neocortex, specifically layer I of the prefrontal and somatosensory cortex, and lays the basis for further investigations including deeper cortical layers or other brain areas, such as the hippocampus [28]. Furthermore, future studies will have to systematically elucidate sex-specific effects of Dsg2 mutations on brain tissue. In humans, there are both evident differences in the manifestation of HF [29] and the occurrence of cognitive dysfunction [30] between sexes.

## Electronic supplementary material

Below is the link to the electronic supplementary material.

Supplementary Material 1

## Data Availability

The datasets used and analyzed during the current study are available from the corresponding authors on reasonable request.

## Abbreviations

CB: Cacodylate buffer
Dsg2, DSG2: Desmoglein-2
HF: Heart failure
PFC: Prefrontal cortex
PSD: Postsynaptic density
ROI: Region of interest
SSC: Somatosensory cortex
TEM: Transmission electron microscopy

## References

[1] Roger VL. Epidemiology of heart failure: a contemporary perspective. Circ Res. 2021;128(10):1421–34. 10.1161/CIRCRESAHA.121.318172. 33983838

[2] Mene-Afejuku TO, Pernia M, Ibebuogu UN, Chaudhari S, Mushiyev S, Visco F, et al. Heart failure and cognitive impairment: clinical relevance and therapeutic considerations. Curr Cardiol Rev. 2019;15(4):291–303. 10.2174/1573403X15666190313112841. PMC8142355 31456512

[3] Schinner C, Xu L, Franz H, Zimmermann A, Wanuske MT, Rathod M, et al. Defective desmosomal adhesion causes arrhythmogenic cardiomyopathy by involving an integrin-alphaVbeta6/tgf-beta signaling cascade. Circulation. 2022;146(21):1610–26. 10.1161/CIRCULATIONAHA.121.057329. PMC9674449 36268721

[4] Elliott PM, Anastasakis A, Asimaki A, Basso C, Bauce B, Brooke MA, et al. Definition and treatment of arrhythmogenic cardiomyopathy: an updated expert panel report. Eur J Heart Fail. 2019;21:955–64. 10.1002/ejhf.1534 PMC6685753 31210398

[5] Li M, Zhang J, Hu Y, Zhang Y. Cognitive impairment in patients with heart failure: physiopathology, clinical implications, and therapeutic considerations. J Cardiol. 2025;85(6):465–72. 10.1016/j.jjcc.2025.02.007. 39952353

[6] Billups B, Forsythe ID. Presynaptic mitochondrial calcium sequestration influences transmission at mammalian central synapses. J Neurosci. 2002;22(14):5840–47. 10.1523/JNEUROSCI.22-14-05840.2002. PMC6757942 12122046

[7] Myeong J, Stunault MI, Klyachko VA, Ashrafi G. Metabolic regulation of single synaptic vesicle exo- and endocytosis in hippocampal synapses. Cell Rep. 2024;43(5):114218. 10.1016/j.celrep.2024.114218. PMC11221188 38758651

[8] Gray EG. Axo-somatic and axo-dendritic synapses of the cerebral cortex: an electron microscope study. J Anat. 1959;93:420–33. PMC1244535 13829103

[9] Lenz M, Eichler A, Kruse P, Muellerleile J, Deller T, Jedlicka P, et al. All-trans retinoic acid induces synaptopodin-dependent metaplasticity in mouse dentate granule cells. Elife. 2021;10. 10.7554/eLife.71983. PMC8560091 34723795

[10] Deller T, Korte M, Chabanis S, Drakew A, Schwegler H, Stefani GG, et al. Synaptopodin-deficient mice lack a spine apparatus and show deficits in synaptic plasticity. Proc Natl Acad Sci USA. 2003;100(18):10494–99. 10.1073/pnas.1832384100. PMC193589 12928494

[11] Wang W, Zhao F, Lu Y, Siedlak SL, Fujioka H, Feng H, et al. Damaged mitochondria coincide with presynaptic vesicle loss and abnormalities in alzheimer’s disease brain. Acta Neuropathol Commun. 2023;11:54. 10.1186/s40478-023-01552-7. PMC10067183 37004141

[12] Junqueira LCU, Montes GS, Krisztán RM. The collagen of the vertebrate peripheral nervous system. Cell Tissue Res. 1979;202(3):453–60. 10.1007/BF00220437. 519713

[13] Reynolds ES. The use of lead citrate at high pH as an electron-opaque stain in electron microscopy. J Cell Biol. 1963;17(1):208–12. 10.1083/jcb.17.1.208. PMC2106263 13986422

[14] Schindelin J, Arganda-Carreras I, Frise E, Kaynig V, Longair M, Pietzsch T, et al. Fiji: an open-source platform for biological-image analysis. Nat Methods. 2012;9:676–82. 10.1038/nmeth.2019. PMC3855844 22743772

[15] Hemkemeyer SA, Quintiliani S, Schaller A, Madkhour R, Elchinova E, Schröder-Schwarz J, et al. A Swiss *DSG2* founder variant promotes left ventricular thrombus formation causing cardioembolic stroke in autosomal recessive arrhythmogenic cardiomyopathy. 2026. medRxiv, 2026.08.17.26359854.

[16] Deswal A, Petersen NJ, Feldman AM, Young JB, White BG, Mann DL. Cytokines and cytokine receptors in advanced heart failure: an analysis of the cytokine database from the vesnarinone trial (VEST). Circulation. 2001;103:2055–59. 10.1161/01.CIR.103.16.2055. 11319194

[17] Tyan CC, Armstrong S, Scholl D, Stirrat J, Blackwood K, El-Sherif O, et al. Stress hypoperfusion and tissue injury in hypertrophic cardiomyopathy: spatial characterization using high-resolution 3-tesla magnetic resonance imaging. Circ Cardiovasc Imag. 2013;6(2):229–38. 10.1161/CIRCIMAGING.112.000170. 23413422

[18] Dejanovic B, Sheng M, Hanson JE. Targeting synapse function and loss for treatment of neurodegenerative diseases. Nat Rev Drug Discov. 2024;23:23–42. 10.1038/s41573-023-00823-1. 38012296

[19] Fuchsberger T, Paulsen O. Modulation of hippocampal plasticity in learning and memory. Curr Opin Neurobiol. 2022;75:102558. 10.1016/j.conb.2022.102558. 35660989

[20] Griffiths J, Grant SGN. Synapse pathology in Alzheimer’s disease. Semin Cell Dev Biol. 2023;139:13–23. 10.1016/j.semcdb.2022.05.028 35690535

[21] Meyer D, Bonhoeffer T, Scheuss V. Balance and stability of synaptic structures during synaptic plasticity. Neuron. 2014;82:430–43. 10.1016/j.neuron.2014.02.031. 24742464

[22] Doherty J, Dingledine R. Regulation of excitatory input to inhibitory interneurons of the dentate gyrus during hypoxia. J Neurophysiol. 1997;77:393–404. 10.1152/jn.1997.77.1.393. 9120580

[23] Hwang S, Ham S, Lee S-E, Lee Y, Lee GH. Hypoxia regulates the level of glutamic acid decarboxylase enzymes and interrupts inhibitory synapse stability in primary cultured neurons. NeuroToxicology. 2018;65:221–30. 10.1016/j.neuro.2017.10.006. 29107683

[24] *Allen Institute for Brain Science. Allen Mouse Brain Atlas [Dsg2 - RP_060518_04_H06 - sagittal]. [Online]. 2011. [Accessed 02.10.2026 2026].Allen Mouse Brain Atlas [Dsg2 - RP_060518_04_H06 - sagittal]* [Online] (2011). https://www.mouse.brain-map.org.

[25] Human Protein Atlas*, *DSG2 [Online] (2026). Available: https://www.proteinatlas.org/ENSG00000046604-DSG2/brain [Accessed 02.10.2026 2026].

[26] Sjöstedt E, Zhong W, Fagerberg L, Karlsson M, Mitsios N, Adori C, et al. An atlas of the protein-coding genes in the human, pig, and mouse brain. Science. 2020;367(6482):eaay 5947. 10.1126/science.aay5947. 32139519

[27] Roy B, Shao X, Jobanputra KJ, Fonarow GC, Wang DJJ, Woo M, et al. Blood-brain barrier dysfunction in adults with heart failure. J Neuroimaging. 2025;35(4):e70068. 10.1111/jon.70068. PMC12236461 40613567

[28] Yang M, Sun D, Wang Y, Yan M, Zheng J, Ren J. Cognitive impairment in heart failure: landscape, challenges, and future directions. Front Cardiovasc Med. 2021;8:831734. 10.3389/fcvm.2021.831734 PMC8858826 35198608

[29] Regitz-Zagrosek V. Sex and gender differences in heart failure. Int J Heart Fail. 2020;2:157–81. 10.36628/ijhf.2020.0004. PMC9536682 36262368

[30] Kuipers S, Kappelle LJ, Greving JP, Amier RP, De Bresser J, Bron EE, et al. Sex differences in cognitive functioning in patients with heart failure. Int J Cardiol. 2025;418:132603. 10.1016/j.ijcard.2024.132603. 39343304

[31] Allen Institute for Brain Science (2011). Allen Reference Atlas - Mouse Brain [Mouse, P56, Coronal]. [Online]. 2005. Available: [Accessed 02.10.2026 2026]. https://www.atlas.brain-map.org. Available: Accessed 02.10.2026 2026.

