# Supplementary material for: Dsg2 arrhythmogenic cardiomyopathy is accompanied by neocortical synaptic changes in adult mice
Source: Mol Brain. 2026 Nov 11;19:81. doi: 10.1186/s13041-026-01355-6 (PMC13757226; doi:10.1186/s13041-026-01355-6)
Supplement: Supplementary file 1 [file 13041_2026_1355_MOESM1_ESM.docx]

**Figure S1: Sirius Red stain of hearts of wildtype (Dsg2^+/+^) and Dsg2 mutant mice (Dsg2^W2A/W2A^).**

Representative Sirius Red stain of hearts at mid-papillary transversal level to highlight biventricular fibrosis in red accompanied by HF in Dsg2^W2A/W2A^ animals.

**Figure S2: Location of analyzed brain regions in the mouse neocortex.**

(A) Representative Nissl stainings from the Allen Mouse Brain Atlas (Allen Institute, (2005)) at the approximate position in the mouse brain, where tissue sections for TEM were taken. Approximate location of semi-thin sections shown in (B) are indicated by red rectangles. Scale bars, 1 mm.
(B) Representative images of semi-thin sections of wildtype brains of the PFC (upper image) and the SSC (lower image). Synapses were analyzed in layer I of the respective region. Scale bars, 200 µm. PFC, prefrontal cortex, SSC, somatosensory cortex.

**Figure S3: Morphological characterization of synapses in the somatosensory cortex.**

(A) TEM sample image of brain tissue around a vessel in the superficial layers of the somatosensory cortex. SSC, somatosensory cortex. Scale bar, 2 µm.

(B)-(C) Sample images and group data of synapses in the superficial layers of the somatosensory cortex in a wildtype (Dsg2^+/+^) and a Dsg2 mutant animal (Dsg2^W2A/W2A^). Analyzed morphological parameters of the presynaptic compartment are indicated with a red overlay (upper panel: presynaptic area; lower panel: synaptic vesicles). For group data, median (M) with the first and third quartiles (in brackets) are provided for each group (n_WT_ = 1426 synapses in one animal, n_W2A_ = 1408 synapses in one animal). Scale bars, 500 nm.

(D)-(E) Sample images and group data of postsynaptic parameters of synapses in the superficial layers of the somatosensory cortex in a wildtype (Dsg2^+/+^) and a Dsg2 mutant animal (Dsg2^W2A/W2A^). Analyzed morphological parameters of the postsynaptic compartment are indicated with a red overlay (upper panel: PSD length; middle panel: postsynaptic area; lower panel: spine apparatus organelle area). For group data, median (M) together with the first and third quartiles (in brackets) are provided for each group (PSD length: n_WT_= 1426 synapses in one animal, n_W2A_= 1408 synapses in one animal; postsynaptic area: n_WT_= 1426 synapses in one animal, n_W2A_= 1408 synapses in one animal, 19 data points are outside the axis limits; spine apparatus area: n_WT_= 349 synapses in one animal, n_W2A_= 265 synapses in one animal). Scale bars, 500 nm.

Mann-Whitney test. Colored dots represent individual data points. Error bars represent median ± interquartile range. ns, no statistically significant difference; *p < 0.05; **p < 0.01.

**Figure S1**

**Figure S2**

**Figure S3**
